# Predictive factors and prevalence of microalbuminuria in HIV-infected patients: a cross-sectional analysis

**DOI:** 10.1186/s12882-017-0672-9

**Published:** 2017-07-28

**Authors:** Katia Falasca, Marta Di Nicola, Italo Porfilio, Claudio Ucciferri, Elisabetta Schiaroli, Chiara Gabrielli, Daniela Francisci, Jacopo Vecchiet

**Affiliations:** 1 Clinic of Infectious Diseases, Department of Medicine and Science of Aging, “G. d’Annunzio” University, School of Medicine, Via dei Vestini, 66013 Chieti, Italy; 2 Laboratory of Biostatistics, Department of Medical, Oral and Biotechnological Sciences, University “G. d’Annunzio”, Chieti- Pescara, Italy; 3 Division of Hygiene, Epidemiology and Public Health, Department of Medicine and Science of Aging, University “G. d’Annunzio”, Chieti-Pescara, Italy; 4 Department of Medicine and Health Sciences, University of Molise, Campobasso, Italy; 5 Clinic of Infectious Diseases, Department of Medicine, University of Perugia, Perugia, Italy

**Keywords:** Kidneys, Adverse events, Antiretroviral therapy

## Abstract

**Background:**

Renal dysfunction is a common problem in the HIV+ population, due to the effect of both the HIV virus and the several classes of ARV drugs such as tenofovir (TDF). It is also known that the presence of renal damage correlates with cardiovascular risk and therefore with the risk of mortality of the patients accordingly. The detection of early renal damage is very important. Albuminuria and microalbuminuria are markers of early kidney disease and cardiovascular risk. The aim of the study is to evaluate the prevalence of microalbuminuria in a large polycentric sample, of unselected and consecutive HIV-patients followed as outpatients, and to assess its association with different therapeutic regimens.

**Methods:**

We studied 326 patients with a mean age of 48.4 ± 1.6 years, treated at the Infectious Diseases Clinics of Chieti and Perugia for 48 weeks. The main metabolic parameters and the microalbuminuria levels in a single sample of urine were evaluated.

**Results:**

Microalbuminuria was detected in 61.0% of patients at T0 and in 49.7% after 48 weeks of observation with a median values of 1.1 mg/L (IQR: 0-2.7) vs. 0 mg/L (IQR: 0-2.0). 70% of the enrolled population did not show changes in microalbuminuria levels over time, 19% showed improvement, and 11% of the population had a worsening of microalbuminuria levels without any alteration of creatinine, uric acid and GFR-MDRD. We also found a statistically significant association between the development of microalbuminuria and gender (*p* < 0.035), Arterial Hypertension (AH) (*p* < 0.028) and therapy with TDF (*p* < 0.050).

**Conclusion:**

We showed a very high prevalence of microalbuminuria, much higher than the literature data; the use of TDF affects the renal function in a statistically significant way and should therefore be considered a risk factor for kidney damage, which can be early assessed with the measurement of microalbuminuria.

## Background

HIV infection is an early atherosclerotic and vascular disease risk factor leading to an increased risk of cardiovascular events in comparison with HIV negative [1]. Currently cardiovascular disease (CVD) is the third cause of death among people infected with HIV in the United States. Similarly, also the renal disease is increasing as a complication of HIV. HIV patients may also show an increased incidence of hypertension, that represents an additional risk factor for cardiovascular and renal disease [2].

HIV patients typically have 10 years a cardiovascular risk higher than non-HIV (DAD) [3]. Evaluation of prognostic scores, such as the Framingham Risk Score and the DAD 5 years Estimated Risk Calculator, combined with rapid execution and evaluation of biochemical markers, and evaluation of the classic cardiovascular risk factors as diabetes, dyslipidemia, smoking, etc. can lead to a better stratification of patients and add predictive factors for acute vascular events or worsening of renal function [4, 5].

Microalbuminuria is a widely recognized early marker of renal dysfunction [6], which can be easily and reliably expressed by urine albumin-to-creatinine ratio (UACR) [7]. Either microalbuminuria or increased UACR have been associated with an increased CV risk in different clinical studies [8]. In addition, microalbuminuria often complicates the course of HIV infection [9], being associated with an increased risk of future CV disease events [10].

Albuminuria is a quantitative measure of the albumin excreted in the urine within 24 h and, as such, requires a rather complex execution procedure; microalbuminuria is an exam of easier implementation in daily practice, being evaluated as the mean of two evaluations of standard samples of urine. The association between albuminuria and cardiovascular risk is well documented in the general population and in HIV-patients [11]. In recent years, the role of microalbuminuria has also been preliminarily evaluated in HIV patients; moreover, extensive data documented the strong correlation between albuminuria and microalbuminuria [9]. The identification of overt proteinuria or even microalbuminuria, might be an important tool for the early detection of chronic kidney disease (CKD) in HIV-infected individuals, and there are several techniques of evaluation for microalbuminuria measurement [12, 13]. Overall microalbuminuria has a prevalence between 19 and 34%, resulting as an important risk factor in the HIV population [14, 15]. The presence of microalbuminuria in relation to different antiretroviral therapy (ART) regimens and, in particular, the use of tenofovir (TDF) was documented in different studies [14, 16, 17].

This study has three objectives. First, it is aimed at determining the period prevalence of microalbuminuria in an HIV-infected population in a polycentric cohort, in order to reach an assessment of the microalbuminuria rate associated with chronic HIV infection per se. Second, it is aimed at evaluating the variability of albuminuria over time, and the positive and negative predictive factors of a single urine specimen for persistent microalbuminuria. Third, its goal is to examine the clinical and laboratory correlates of microalbuminuria in this population, particularly with ART.

## Methods

### Study design

This is a multicenter, prospective, observational study, with a cross-sectional assessment at baseline and after 48 weeks, with longitudinal data collection on microalbuminuria by single urine sample. Three hundred twenty-six patients were enrolled and were subjected to an evaluation of renal function with a urine analysis and microalbuminuria assessment. The volume of glomerular filtrate was then calculated using the “Modification of Diet in Renal Disease-4” – MDRD-4 - equation. This assessment was repeated after 48 weeks, to define T0 and T48.

Patients were enrolled from December 2014 to December 2015. We consecutively enrolled in this cross-sectional study 326 HIV-infected patients on ART, with no evidence of kidney disease, who were being referred to the Day Hospital of Infectious Diseases, Department of Medicine and Science of Ageing, “G. d’Annunzio” University (Chieti-Pescara, Italy) and to the Day Hospital of Infectious Diseases Department of Medicine, University of Perugia (Perugia, Italy). Exclusion criteria included: age under 18 years, current pregnancy, an estimated Glomerular Filtration Rate (eGFR) < 60 ml/min, calculated using the MDRD-4, transient causes of physiologic albuminuria (e.g. fever, exercise), out of range urinary specific gravity values, acute pathologic conditions and opportunistic infections within the past 3 months, organ transplants, recent interferon or direct-acting antiviral (DAA) therapies and abuse of alcohol (greater than or equal to 80 g/day), and/or drugs in the past 6 months. Data regarding social and demographic issues, comorbidities (hepatitis HCV, hypertension and diabetes), viro-immunological profile, current medications, current ART were collected. No HIV-HCV coinfected patients received an anti-HCV therapy during the study period.

### Biochemical analyses

Overnight fasting venous blood samples were collected during the intervention period for immunological analysis and evaluation of renal function and microalbuminuria at T0 and T48. Collection of the main demographic data: age, gender, race, risk factor, smoke; of clinical data (weight, height, blood pressure, years of infection, the presence of co-infection HCV); and of laboratory parameters (liver function, glucose profile, lipid profile).

Microalbuminuria was measured on single-day urine samples using particle-enhanced immunonephelometric assays (BN II System, Siemens Healthcare Diagnostics, Inc). The inter-assay coefficient of variation was ≤8% and the he intra-assay coefficient of variation was ≤6.3%. In this study, microalbuminuria was defined as a urinary albumin excretion rate greater than 1 mg/dL, determined over two assessments, the first of which confirmed by the presence of protein excretion. The immunonephelometric technique allowed to quickly and simply run the analysis, despite the technical limits in the given clinical environment. This technique has been validated for population and first level screening for albumin analysis [18, 19]. The serum creatinine was measured with an enzymatic assay (Ortho Clinical Diagnostics) with a normal range of 0.66-1,25 mg/dl. The intra-assay and inter-assay coefficients of variation (CV) were <3%.

Finally a study on cystatin C was performed for the assessment of cardiovascular risk and for correlation with the levels of microalbuminuria. The measurements of serum cystatin C were done on fresh fasting blood samples using a BN II nephelometric system (Siemens Healthcare Diagnostics, Deerfield, Illinois, USA), with a particle-enhanced immunonephelometric assay.

Routine laboratory tests were performed at the Divisions of Clinical Pathology in the hospital of Chieti and Perugia.

### Virologic and immunologic markers

CD4+- and CD8+-T cell counts were obtained by flow cytometry of lymphocyte subpopulations. Plasma viral load (HIV-RNA) was determined using the “Amplicor” method (Roche Molecular Diagnostics, Milan, Italy) with a detection limit of >20 HIV RNA copies/mL of plasma.

### Statistical evaluation

The qualitative variables were summarized as frequencies and percentages. The quantitative variables were summarized as mean and standard deviation. The results were reported separately for each of three subgroups (equal, improved and worse). Shapiro-Wilk test was performed to verify normal distribution.

Patients were divided according to the presence/absence of microalbuminuria at time 0 and at time 48 and were divided into 3 subgroups: the group of patients with no changes in the microalbuminuria status in the 48 weeks of study was defined as “equal”; the group of patients that overcame the microalbuminuria in the 48 weeks of study was defined as “Improved” and the group of patients that had a worsening of microalbuminuria in the 48 weeks of study was defined as “Worse”.

Statistical differences in three subgroups were evaluated applying chi-square test for qualitative variables and Student t test for unpaired data for quantitative variables.

Metabolic parameters were analyzed using different linear mixed models for repeated data. This approach allows explicit modelling of the within-person and between-person variation in the outcome, while taking into account the correlation between repeated measurements on the same individual.

The linear mixed model for repeated measurements was used to regress measures at T0 and T48 on the fixed-effect factors assuming unstructured covariance matrix and to evaluate the effect of each factor (time and subgroup) and their interaction on metabolic parameters.

The statistical significance was assumed at *p* ≤ 0.05.

Data was analyzed by the SPSS® Advanced Statistical ™ 13 software (2004, Chicago, IL, USA).

## Results

A total of 326 patients were enrolled, 261 (80.1%) of which were males, with a mean age of 48.4 ± 11.6 years, 300 of them were of Caucasian ethnicity. The risk factors for HIV infection were: heterosexual transmission 154 (47.2%), men sex with men 120 (36.8%) and 49 (15.0%) history of previous intravenous drug use (Table 1). The average of infection was 10 (IQR: 5.0-17.0) years. Two hundred ninety-seven patients (91.1%) were in ART without therapeutic changes for more than 12 months, 178 (54.6%) of which were under treatment with TDF. HIV-RNA was negative in 254 patients (77.9%) at T0 and 312 (97.7%) patients had a negative viremia at T48, and a CD4 cell count >300 cell/ml. Furthermore they had normal Body mass index (BMI) with mean of 24.8 (22.3-28.0). Sixty-six patients (20.2%) had a HCV-related chronic hepatitis, 30 (9.2%) patients were diabetic, 89 (27.3%) had a diagnosis of hypertension, 73 (22.3%) patients had a dyslipidemia.

**Table 1:** Baseline patient’s characteristics according to subgroup. Data are expressed as frequency and percentage or as median (IQR)

*Variables*	Overall	Equal	Improved	Worse	*p-value* ^*a*^
Gender					**0.035**
Female	65 (19.9)	40 (17.5)	12 (19.4)	13 (36.1)	
Male	261 (80.1)	188 (82.5)	50 (80.6)	23 (63.9)	
Race					0.522
caucasian	300 (92.0)	211 (92.5)	55 (88.7)	34 (94.4)	
non caucasian	26 (8.0)	17 (7.5)	7 (11.3)	2 (5.6)	
Risk factor					0.213
homosexuals	120 (36.8)	90 (40.0)	22 (35.5)	8 (22.2)	
IV Drug user	49 (15.0)	32 (14.2)	12 (19.3)	5 (13.9)	
heterosexuals	154 (47.2)	103 (45.8)	28 (45.2)	23 (63.9)	
HCV					0.488
no	360 (79.7)	182 (79.8)	47 (75.8)	31 (86.1)	
yes	66 (20.3)	46 (20.2)	15 (24.2)	5 (13.9)	
Diabetes					0.242
no	296 (90.8)	203 (89.0)	59 (95.2)	34 (94.4)	
yes	30 (9.2)	25 (11.0)	3 (4.8)	2 (5.6)	
Hypertension					**0.028**
no	237 (72.7)	156 (68.4)	52 (83.9)	29 (80.6)	
yes	89 (27.3)	72 (31.6)	10 (16.1)	7 (19.4)	
ART					0.415
no	25 (7.7)	20 (8.8)	4 (6.3)	1 (2.8)	
yes	301 (92.3)	207 (92.2)	59 (93.7)	35 (97.2)	
TDF based ART					**0.040**
no	123 (45.4)	94 (45.4)	19 (32.2)	10 (28.6)	
yes	178 (54.6)	113 (54.6)	40 (67.8)	25 (71.4)	
Type of ART					0.926
NNRTI	146 (47.0)	101 (47.4)	29 (48.3)	16 (43.2)	
IP	120 (38.8)	81 (38.0)	24 (40.0)	15 (40.5)	
INI	44 (14.2)	31 (14.6)	7 (11.7)	6 (16.3)	
AIDS					0.544
no	247 (75.8)	169 (74.1)	50 (80.6)	28 (77.8)	
yes	79 (24.2)	59 (25.9)	12 (19.4)	8 (22.2)	
Smoke					0.609
no	153 (46.9)	108 (47.4)	28 (45.2)	17 (47.2)	
yes	130 (39.9)	88 (38.6)	29 (46.8)	13 (36.1)	
ex	43 (13.2)	32 (14.0)	5 (8.0)	6 (16.7)	
BMI	24.8 (22.3–28.0)	24.6 (22.1-27.7)	26.0 (23.6-30.1)	24.1 (22.5-26.7)	**0.024** ^**b**^
Duration of disease	10.0 (5.0-17.0)	11.0 (6.0-17.7)	11.0 (4.0-17.0)	7.0 (5.0-15.7)	0.302^b^

^a^Chi-squared test; ^b^Student t-test for unpaired data. *P values* in bold suggest significant values.

**Fig. 1:**
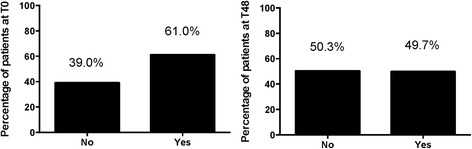
Prevalence of microalbuminuria at T0 and T48

The prevalence of microalbuminuria was found to be 61.0% at T0, and declined to 49.7% at T48 (Fig. 1) and the median microalbuminuria values decreased from 1.1 mg/L (IQR: 0-2.7) at T0 to 0 mg/L (IQR: 0-20) at T48.

**Fig. 2:**
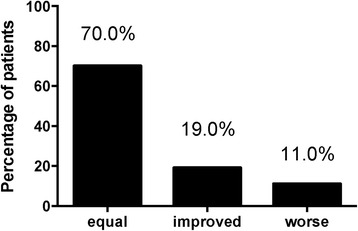
Prevalence of microalbuminuria in three subgroups. Equal: group of patients that no changes the microalbuminuria status in the 48 weeks of study. Improves: group of patients that resolved the microalbuminuria status in the 48 weeks of study. Worse: group of patients that has a worsening of microalbuminuria status in the 48 weeks of study

By splitting the patients into 3 subgroups (Equal, Improved and Worse), we showed that during the study period there was no change in the presence of microalbuminuria in 70.0% of the cases, we found an improvement in 19.0% of patients, and a worsening in 11.0% of them (Fig. 2). The Equal group had median levels of microalbuminuria of 1.2 mg/L (IQR: 0-2.9) at T0 and 1.2 mg/L (IQR: 0-2.8) at T48; the Improved group had levels of microalbuminuria of 1.5 mg/L (IQR: 1.1-2.4) at T0 vs 0 mg/L (IQR: 0-0) at T48 (*p* < 0.001); the Worse group had levels of microalbuminuria of 0 mg/L (IQR: 0-0) at T0 vs 1.5 mg/L (IQR: 1.2-1.8) at T48 (*p* < 0.001).

Based on this classification in Equal, Improved and Worse, we found a statistically significant association between the development of microalbuminuria and gender (*p* = 0.035), Arterial Hypertension (AH) (*p* = 0.028), BMI (*p* = 0.024) and therapy with TDF (*p* = 0.040). Among the patients receiving ART, 54.6% were treated with TDF. Dividing the population into three subgroups we showed that 71.4% of the patients whose microalbuminuria levels got worse (Worse group) used therapy with TDF (*p* = 0.040). The use of NNRTI, IP or INI did not influence the variation of microalbuminuria (Table 1).

The Improved and Worse subgroup had a significant increase in the cholesterol levels, regardless of the interaction of different subgroups (*p* = 0.020). The Improved subgroup had a significant increase in the LDL-cholesterol, regardless of time and of type of group (*p* = 0.009 and *p* = 0.007). Blood glucose, creatinine, uric acid, MDRD-GFR, serum albumin and transaminases have not changed in the three groups remaining in the normal range. Finally there was an increase in the number of CD4+ cells up to T48 in all the three groups which is statistically significant over time (*p* = 0.001).

Another interesting element was the increase of Cystatin C levels during time (*p* < 0.001), which is equal in the 3 subgroups (Table 2).

**Table 2:** Mean and standard deviation of metabolic parameters

	Equal		Improved		Worse		Time^a^	Subgroup^b^	Interaction^c^
	T0	T48	T0	T48	T0	T48			
Systolic blood pressure, mmHg	125.0 ± 14.9	125.4 ± 14.0	122.8 ± 15.4	124.6 ± 16.1	121.4 ± 17.3	123.1 ± 15.8	0.097	0.432	0.534
Diastolic blood pressure, mmHg	78.0 ± 9.6	78.5 ± 12.1	77.2 ± 8.9	78.8 ± 10.2	75.8 ± 9.9	77.0 ± 9.7	0.114	0.557	0.684
Total cholesterol, mg/dL	190.8 ± 43.2	191.6 ± 42.4	182.7 ± 47.7	203.3 ± 54.3	187.3 ± 51.8	204.9 ± 44.9	0.141	0.646	**0.020**
Triglycerides, mg/dL	159.5 ± 90.2	167.2 ± 145.9	146.5 ± 81.6	142.0 ± 74.6	136.1 ± 70.4	142.4 ± 98.2	0.958	0.384	0.980
LDL-cholesterol, mg/dL	111.1 ± 37.1	114.0 ± 33.6	103.7 ± 41.4	125.9 ± 38.9	119.1 ± 47.3	115.4 ± 35.8	**0.009**	0.872	**0.007**
Fasting glucose, mg/dL	92.5 ± 21.3	91.7 ± 30.3	91.5 ± 20.7	81.9 ± 9.2	96.4 ± 25.6	89.3 ± 15.8	0.176	0.117	0.257
CD4, %	30.2 ± 9.8	37.1 ± 54.3	28.1 ± 10.9	29.2 ± 10.5	32.1 ± 6.3	33.2 ± 7.9	0.514	0.581	0.748
CD4+, cell/μL	651.4 ± 295.8	688.0 ± 294.7	659.5 ± 344.0	767.1 ± 398.1	663.0 ± 281.2	755.4 ± 323.4	**0.001**	0.675	0.376
CD4/CD8 ratio	0.8 ± 0.5	0.9 ± 0.4	0.8 ± 0.5	0.8 ± 0.4	0.9 ± 0.3	1.0 ± 0.4	0.208	0.699	0.178
Creatinine, mg/dL	0.9 ± 0.2	0.9 ± 0.3	0.8 ± 0.2	0.9 ± 0.2	0.8 ± 0.1	0.8 ± 0.1	0.600	0.359	0.503
Uric acid, mg/dL	5.3 ± 2.0	5.3 ± 1.5	5.1 ± 1.2	5.1 ± 1.5	5.3 ± 2.3	4.8 ± 0.9	0.656	0.608	0.733
MDRD glomerular filtration rate, mL/min/1.73 m2	100.7 ± 24.4	101.6 ± 23.4	89.0 ± 55.2	90.5 ± 27.6	95.7 ± 17.3	91.9 ± 16.5	0.991	0.728	0.870
Albuminemia, g/dl	4.4 ± 0.4	4.4 ± 0.3	4.3 ± 0.4	4.4 ± 0.4	4.4 ± 0.3	4.5 ± 0.3	0.217	0.487	0.725
GOT/GPT ratio	0.8 ± 0.3	0.9 ± 0.3	0.9 ± 0.4	0.9 ± 0.3	1.0 ± 0.3	1.2 ± 0.7	**0.004**	**0.021**	**0.060**
Cystatin C, mg/L	0.8 ± 0.2	0.9 ± 0.2	0.8 ± 0.2	0.9 ± 0.2	0.7 ± 0.1	0.8 ± 0.1	**<0.001**	0.612	0.250

^a^Probability that values are influenced by time. For each variable, the difference has been tested between the mean of the T0 and the mean of the T48, independently of subgroup

^b^Probability that values are influenced by the change of state of microalbuminuria. For each variable, the difference has been tested between the means of the subgroups, independently of time

^c^Probability that the effect of the treatment time is different in the three subgroups (equal, improved and worse)

*p* values in bold suggest significant values

## Discussion

The aim of the study was to evaluate the prevalence of microalbuminuria in a large sample of HIV-positive patients followed as outpatients by using a single- day urine sample. In this study the prevalence of positivity for microalbuminuria in the urinary test was high, from 61 to 49.7%, and we managed to show a decreasing trend in the levels of microalbuminuria observed during the 48-weeks of observation, probably because of the criterion used for the diagnosis of microalbuminuria that allows to highlight even early alterations. The prevalence of microalbuminuria in HIV+ population was estimated to be ranging from 50 to 58% [9, 20, 21]. In another cohort of HIV+ patients, these values were three to five times lower than in this study and compared to the general population [22]. Hadigan et coll. Conducted a study to determine the presence of microalbuminuria in HIV+ population, reporting a prevalence of 14% [9]. Other authors reported a prevalence of microalbuminuria of 19.4, 29.8 and 31.6%, respectively among patients without hypertension or evidence of other renal diseases [23, 24], these evaluations had different cut-offs and were made with different more expensive and complexe methods.

In a multivariate analysis, systemic systolic pressure, serum beta-2 globulin and duration of infection with HIV were found to be independent markers of microalbuminuria [22]. In this study the results showed the influence of gender, presence of hypertension, high BMI and use of TDF with microalbuminuria, but not with other comorbidity, such as diabetes or smoke.

By subdividing the population into three groups, “Equal status”, “Improved status” and “Worse status” with respect to microalbuminuria, a particularly interesting data was that 11.1% of patients in 48 weeks had a worsening of microalbuminuria levels, and the 71.4% of them were undergoing TDF. This data was well documented in literature, in fact the microalbuminuria was associated with the use TDF as a marker of early renal failure [16, 25, 26].

Antiretroviral therapy was reported to be able to contribute to kidney injury either directly, by inducing acute tubular necrosis, nephropathy by crystals deposit-nephropaty of or renal tubular disorders or indirectly through pharmacologic interactions [27].

The first case of TDF-induced acute kidney disease was published in 2001. Several cases were reported since that time and it has now been determined that the use of TDF presents risks for tubular toxicity [27]. Some facilitators have been identified, such as the co-prescribing of didanosine, a pre-existing chronic renal failure, a low body weight and an associated diabetes mellitus. Conversely, whether a long-term use of TDF can be a cause of renal toxicity is still debated. Some studies suggest a decrease of GFR when TDF is prescribed for a long period of time, while others indicate that TDF is safe for kidneys, even after many years of administration [28].

In order to verify whether the TDF-induced renal damage is due to a selective engagement of the proximal tubule mitochondria, the following parameters were evaluated: urinary markers of mitochondrial toxicity (cytochrome C), cytosolic toxicity (glutathione S-transferase alpha) along with classical indicators of renal function. The levels of cytochrome C were significantly higher at 1 and 6 months after the shift to non-thymidine regimens in patients receiving TDF/FTC. The other parameters were normal. The study suggests a subclinical mitochondrial damage associated with TDF [29]. The authors recorded also low percentages of patients with co-morbidities such as diabetes, hypertension and dyslipidemia so it is likely that the kidney damage and cardiovascular risk are related to both the use of ART and the action of the virus itself [30].

Patients with HIV infection frequently show a renal disease secondary to the infection itself. Cooper et al. estimated that about 30% of these patients had kidney diseases [31]. The chances of renal disease increase with risk factors such as infection with hepatitis C [32], and/or B, hypertension, diabetes mellitus and dyslipidemia, low white blood cell count CD4 [33–37]. In this study the microalbuminuria was associated only with male gender, hypertension and BMI, but not with diabetes and any lipid alteration.

In recent years, studies have focused on the quest for an alternative indicator of renal function since creatinine suffers from several limitations, such as inter-individual variability and volatility related to extra-renal factors such as age, nutrition, hydration status and the ground muscle. Furthermore creatinine levels increases only the renal function is severely impaired. On the other hand the classic microalbuminuria uses the urine samples on 24 h, a complex examination that requires compliance by the patient, and it is expensive. In this study it was possible to measure the microalbuminuria levels on a single–day urine sample, a simple examination, that requires low compliance by the patient and it is cheaper [38, 39].

We noticed an increase in Cystatin C in all the patients after a 48 weeks observational period and this could relate to a worsened inflammatory state and an increased cardiovascular risk. Yet, the “worse” group shows a more pronounced increase (even if not statistically significant), presumably because of a coupled increase in microalbuminuria and a worsened renal function**.** This biomarker has a constant rate of endogenous production. The independence from muscle mass, along with the extracellular distribution, the exclusive excretion by glomerular filtration and its short half-life, would make it, in theory, a better marker than creatinine. Based on shorter half-life than creatinine (2 h), changes in serum Cystatin C could reflect earlier changes in GFR. To assess renal function, the blood levels of Cystatin C may be used as such, or in the form of equations for the estimation of GFR, applied in the clinical practice with still non-univocal results [40, 41].

Finally microalbuminuria and Cystatin C may represent biomarkers of easy execution, especially for the early evaluation of cardiovascular risk in this population of HIV positive patients [40].

## Conclusion

In conclusion, the data emerging from our study is the increased levels of Cystatin C in all three groups, in particular in HIV+ subjects whose level of microalbuminuria worsens over time and the handiness of measurement of microalbuminuria and Cystatin C.

The measurement of microalbuminuria by the assay on a single-day urine sample allows to have a simple execution marker for the early individuation of patients with impaired renal function, in particular, those that use of tenofovir.

## Abbreviations

ART: Antiretroviral therapy
BMI: Body mass index
CKD: Chronic kidney disease
CVD: Cardiovascular disease
DAA: Direct-acting antiviral
DAD: Data collection on adverse events of anti-HIV drugs
eGFR: Glomerular filtration rate
IA: Arterial hypertension
INI: Integrase inhibitor
IQR: Interquartile range
MDRD: Modification of diet in renal disease
NNRTI: Non-nucleoside reverse transcriptase inhibitor
NRTI: Nucleoside reverse transcriptase inhibitors
PI: Protease inhibitor
TDF: Tenofovir
UACR: Urine albumin-to-creatinine ratio

## References

[1] Friis-Moller N, Thiebaut R, Reiss P, Weber R, Monforte AD, De Wit S, El-Sadr W, Fontas E, Worm S, Kirk O (2010). Predicting the risk of cardiovascular disease in HIV-infected patients: the data collection on adverse effects of anti-HIV drugs study. Eur J Cardiovasc Prev Rehabil.

[2] Phair J, Palella F (2011). Renal disease in HIV-infected individuals. Curr Opin HIV AIDS.

[3] Friis-Moller N, Weber R, Reiss P, Thiebaut R, Kirk O, d'Arminio Monforte A, Pradier C, Morfeldt L, Mateu S, Law M (2003). Cardiovascular disease risk factors in HIV patients--association with antiretroviral therapy. Results from the DAD study. AIDS.

[4] Mateen FJ, Kanters S, Kalyesubula R, Mukasa B, Kawuma E, Kengne AP, Mills EJ (2013). Hypertension prevalence and Framingham risk score stratification in a large HIV-positive cohort in Uganda. J Hypertens.

[5] Bergersen BM, Sandvik L, Bruun JN, Tonstad S (2004). Elevated Framingham risk score in HIV-positive patients on highly active antiretroviral therapy: results from a Norwegian study of 721 subjects. Eur J Clin Microbiol Infect Dis.

[6] Glassock RJ (2010). Is the presence of microalbuminuria a relevant marker of kidney disease?. Curr Hypertens Rep.

[7] Lemley KV, Abdullah I, Myers BD, Meyer TW, Blouch K, Smith WE, Bennett PH, Nelson RG (2000). Evolution of incipient nephropathy in type 2 diabetes mellitus. Kidney Int.

[8] Wang Y, Yuan A, Yu C (2013). Correlation between microalbuminuria and cardiovascular events. Int J Clin Exp Med.

[9] Hadigan C, Edwards E, Rosenberg A, Purdy JB, Fleischman E, Howard L, Mican JM, Sampath K, Oyalowo A, Johnson A (2013). Microalbuminuria in HIV disease. Am J Nephrol.

[10] Schneider A, Jardine AG, Schneider MP, Holdaas H, Holme I, Fellstroem BC, Zannad F, Schmieder RE, Group AS (2013). Determinants of cardiovascular risk in haemodialysis patients: post hoc analyses of the AURORA study. Am J Nephrol.

[11] Choi EK, Shen MJ, Han S, Kim D, Hwang S, Sayfo S, Piccirillo G, Frick K, Fishbein MC, Hwang C (2010). Intrinsic cardiac nerve activity and paroxysmal atrial tachyarrhythmia in ambulatory dogs. Circulation.

[12] Szczech LA, Gupta SK, Habash R, Guasch A, Kalayjian R, Appel R, Fields TA, Svetkey LP, Flanagan KH, Klotman PE (2004). The clinical epidemiology and course of the spectrum of renal diseases associated with HIV infection. Kidney Int.

[13] Wyatt CM, Morgello S, Katz-Malamed R, Wei C, Klotman ME, Klotman PE, D'Agati VD (2009). The spectrum of kidney disease in patients with AIDS in the era of antiretroviral therapy. Kidney Int.

[14] Mpondo BC, Kalluvya SE, Peck RN, Kabangila R, Kidenya BR, Ephraim L, Fitzgerald DW, Downs JA (2014). Impact of antiretroviral therapy on renal function among HIV-infected Tanzanian adults: a retrospective cohort study. PLoS One.

[15] Campbell LJ, Dew T, Salota R, Cheserem E, Hamzah L, Ibrahim F, Sarafidis PA, Moniz CF, Hendry BM, Poulton M (2012). Total protein, albumin and low-molecular-weight protein excretion in HIV-positive patients. BMC Nephrol.

[16] Maggi P, Montinaro V, Bellacosa C, Pietanza S, Volpe A, Graziano G, Strippoli GF, Angarano G (2012). Early markers of tubular dysfunction in antiretroviral-experienced HIV-infected patients treated with tenofovir versus abacavir. AIDS Patient Care STDs.

[17] Giacomet V, Erba P, Di Nello F, Coletto S, Vigano A, Zuccotti G (2013). Proteinuria in paediatric patients with human immunodeficiency virus infection. World J Clin Cases.

[18] Vogt L, Navis G, Koster J, Manolis AJ, Reid JL, de Zeeuw D, Angiotensin IIRATMiISHSG (2005). The angiotensin II receptor antagonist telmisartan reduces urinary albumin excretion in patients with isolated systolic hypertension: results of a randomized, double-blind, placebo-controlled trial. J Hypertens.

[19] Morimoto S, Yano Y, Maki K, Sawada K (2006). Renal and vascular protective effects of telmisartan in patients with essential hypertension. Hypertens Res.

[20] Shabbal DM, Jamda MA, Dalhatu IT, Abdulrahman MB, Isichei C (2014). Comparison of microalbuminuria among treatment naive HIV sero-positive and negative adult clients in faith alive foundation hospital, Jos. Niger Med J.

[21] Masimango MI, Sumaili EK, Jadoul M, Wallemacq P, Mubagwa DK, Makulo RJ, Lepira FB, Nseka NM (2014). Prevalence of microalbuminuria and diagnostic value of dipstick proteinuria in outpatients from HIV clinics in Bukavu, the Democratic Republic of Congo. BMC Nephrol.

[22] Baekken M, Os I, Sandvik L, Oektedalen O (2008). Microalbuminuria associated with indicators of inflammatory activity in an HIV-positive population. Nephrol Dial Transplant.

[23] Kabanda A, Vandercam B, Bernard A, Lauwerys R, van Ypersele de Strihou C (1996). Low molecular weight proteinuria in human immunodeficiency virus-infected patients. Am J Kidney Dis.

[24] Szczech LA, Grunfeld C, Scherzer R, Canchola JA, van der Horst C, Sidney S, Wohl D, Shlipak MG (2007). Microalbuminuria in HIV infection. AIDS.

[25] Vigano A, Zuccotti GV, Martelli L, Giacomet V, Cafarelli L, Borgonovo S, Beretta S, Rombola G, Mora S (2007). Renal safety of tenofovir in HIV-infected children: a prospective, 96-week longitudinal study. Clin Drug Investig.

[26] Overton ET, Patel P, Mondy K, Bush T, Conley L, Rhame F, Kojic EM, Hammer J, Henry K, Brooks JT (2012). Cystatin C and baseline renal function among HIV-infected persons in the SUN study. AIDS Res Hum Retroviruses.

[27] Kalyesubula R, Perazella MA (2011). Nephrotoxicity of HAART. AIDS Res Treat.

[28] Tourret J, Deray G, Isnard-Bagnis C (2013). Tenofovir effect on the kidneys of HIV-infected patients: a double-edged sword?. J Am Soc Nephrol.

[29] Ryom L, Mocroft A, Kirk O, Worm SW, Kamara DA, Reiss P, Ross M, Fux CA, Morlat P, Moranne O (2013). Association between antiretroviral exposure and renal impairment among HIV-positive persons with normal baseline renal function: the D:a:D study. J Infect Dis.

[30] Bergersen BM, Sandvik L, Dunlop O, Birkeland K, Bruun JN (2003). Prevalence of hypertension in HIV-positive patients on highly active retroviral therapy (HAART) compared with HAART-naive and HIV-negative controls: results from a Norwegian study of 721 patients. Eur J Clin Microbiol Infect Dis.

[31] Choi AI, Li Y, Deeks SG, Grunfeld C, Volberding PA, Shlipak MG (2010). Association between kidney function and albuminuria with cardiovascular events in HIV-infected persons. Circulation.

[32] Peters L, Grint D, Lundgren JD, Rockstroh JK, Soriano V, Reiss P, Grzeszczuk A, Sambatakou H, Mocroft A, Kirk O (2012). Hepatitis C virus viremia increases the incidence of chronic kidney disease in HIV-infected patients. AIDS.

[33] Mocroft A, Lundgren JD, Ross M, Fux CA, Reiss P, Moranne O, Morlat P, Monforte A, Kirk O, Ryom L (2016). Cumulative and current exposure to potentially nephrotoxic antiretrovirals and development of chronic kidney disease in HIV-positive individuals with a normal baseline estimated glomerular filtration rate: a prospective international cohort study. Lancet HIV.

[34] Ucciferri C, Falasca K, Mancino P, Di Iorio A, Vecchiet J (2012). Microalbuminuria and hypertension in HIV-infected patients: a preliminary study of telmisartan. Eur Rev Med Pharmacol Sci.

[35] Ucciferri C, Falasca K, Mancino P, Pizzigallo E, Vecchiet J (2011). Proteinuria in an African HIV-infected patient: effects of telmisartan. Infez Med.

[36] Ucciferri C, Falasca K, Vignale F, Di Nicola M, Vecchiet J (2015). Long term effect of telmisartan in HIV-positive male patients with high blood pressure. Braz J Infect Dis.

[37] Vecchiet J, Ucciferri C, Falasca K, Mancino P, Di Iorio A, De Caterina R (2011). Antihypertensive and metabolic effects of telmisartan in hypertensive HIV-positive patients. Antivir Ther.

[38] Ng WY, Lui KF, Thai AC (2000). Evaluation of a rapid screening test for microalbuminuria with a spot measurement of urine albumin-creatinine ratio. Ann Acad Med Singap.

[39] Parikh CR, Fischer MJ, Estacio R, Schrier RW (2004). Rapid microalbuminuria screening in type 2 diabetes mellitus: simplified approach with Micral test strips and specific gravity. Nephrol Dial Transplant.

[40] Falasca K, Ucciferri C, Mancino P, Di Iorio A, Vignale F, Pizzigallo E, Vecchiet J (2010). Cystatin C, adipokines and cardiovascular risk in HIV infected patients. Curr HIV Res.

[41] de Geus HR, Woo JG, Wang Y, Devarajan P, Betjes MG, le Noble JL, Bakker J (2011). Urinary Neutrophil Gelatinase-associated Lipocalin measured on admission to the intensive care unit accurately discriminates between sustained and transient acute kidney injury in adult critically ill patients. Nephron Extra.

